# Diagnosis of Acute Toxoplasmosis by IgG and IgM Antibodies and IgG Avidity in Pregnant Women from Mashhad, Eastern Iran

**Published:** 2019

**Authors:** Karam SHARIFI, Bibi Razieh HOSSEINI FARASH, Fatemeh TARA, Azad KHALEDI, Karim SHARIFI, Seyed Ali Akbar SHAMSIAN

**Affiliations:** 1. Department of Parasitology and Mycology, Faculty of Medicine, Mashhad University of Medical Sciences, Mashhad, Iran; 2. Research Center of Skin Diseases and Cutaneous Leishmaniasis, School of Medicine, Mashhad University of Medical Sciences, Mashhad, Iran; 3. Department of Obstetrics and Gynecology, Mashhad University of Medical Sciences, Mashhad, Iran; 4. Infectious Diseases Research Center, Department of Microbiology and Immunology, Faculty of Medicine, Kashan University of Medical Sciences, Kashan, Iran; 5. Department of Microbiology and Immunology, Faculty of Medicine, Kashan University of Medical Sciences, Kashan, Iran; 6. Iranian Academic Center for Education, Culture, and Research (ACECR), Mashhad, Iran

**Keywords:** IgG avidity, Pregnant women, Toxoplasmosis, Iran

## Abstract

**Background:**

We aimed to evaluate the diagnosis of acute toxoplasmosis by IgG avidity test in pregnant women.

**Methods:**

In this cross-sectional study, 250 blood samples were collected from pregnant women with the first month of their pregnancy referring to health centers of University in Mashhad during 2016. Samples were centrifuged at 3000 rpm for 5 min for separation of serum and were kept in the –20 until use. To detection of acute and chronic toxoplasmosis, anti-*Toxoplasma* antibodies (IgG and IgM, and IgG avidity tests were performed using ELISA. Then, data analyzed using SPSS software by Frequency, Pearson Chi-Square, Likelihood Ratio, and Exact tests. And *P*<0.05 was statistically considered as significant.

**Results:**

Total prevalence of IgG and IgM was 23.2% and 7.2%, respectively. A significant correlation was observed between the mean age and IgG level (*P*<0.05). It was not found any correlation between the history of raw meat consumption, cats keeping, education, and residency site. Moreover, 16 people (6.4%) had IgM antibody, of which, 10 cases (62.5%) with low avidity for IgG and 1 people (6.2%) with moderate avidity and 5 cases (31.3%) with high avidity for IgG. Moreover, 76% of pregnant women were seronegative.

**Conclusion:**

More than half of the women (62.5%) with positive IgM antibody in their serum had a low avidity for IgG which revealed an acute infection among pregnant women. *Toxoplasma* infection should be considered as an important factor that affects the pregnancy and IgG avidity as an important test for screening the women who need the treatment.

## Introduction

Toxoplasmosis is a protozoan infection with worldwide prevalence. This infection is zoonotic between humans and animals. More than one-third of the world's population is infected with this parasite (1). Toxoplasmosis after salmonellosis and listeriosis is the third most common cause of food-borne infectious deaths (2).

Its seroprevalence is high in countries with high raw meat consumption such as France (54%) and in tropical areas of Latin American, or in African sub-Sahara countries in which cats are abundant and climate is favorable for survival of oocytes (3, 4). In the United States, 51% of women of childbearing age (44–51 yr) are infected with congenital toxoplasmosis and estimated 400–4000 cases per year (2). The prevalence in Canada among women of reproductive age is between 20%–40% and in North of Canada has been reported 59.8% (5). The prevalence of *Toxoplasma* is different in various parts of Iran, varying from 20%–35% in southern warm and dry conditions to 72 ºC in the temperate regions of the north (6).

The three main ways of transmission of toxoplasmosis are through consumption of raw or semi-cooked meat, contact with infected cat-feces with the oocyte, and vertical transmission. During pregnancy, the most common route of contamination is through raw consumption or uncooked meat or contaminated water, or contact with soil (gardening without gloves) or cat. Transmission of toxoplasmosis rarely takes place through blood transfusion and organ transplantation (liver, heart, lung, kidney, and pancreas), too (2, 7).

Toxoplasmosis is linked to the immune system. Therefore, the clinical forms of this disease are different in people with the normal immune system and people with immunodeficiency. The most noticeable clinical symptoms in affected patients are lymphadenopathy and ocular lesions, but in 90% of affected people, no specific clinical symptoms are observed (8). One of the most important cases in this disease is its transmission by the placenta to the fetus in pregnant women. Complications to the embryo can be different from neurological lesions to the chorioretinitis, or it can even emerge years after birth (9). If the mother is infected before pregnancy and does not has an acute infection, an embryo is immune to this disease, while infection of the mother during childbearing can have serious risks (10).

The use of serologic tests is the primary method for the diagnosis of specific *T. gondii* antibody. Usually, to show if a person has been infected in the past, or has recently been infected; a combination of serological tests be required (7).

Unfortunately, the classical serological methods are routinely used for diagnosis are not useful for differentiating between the recent or past toxoplasmosis. Unlike many other infections, primary toxoplasmosis cannot be recognized based on specific IgM of *Toxoplasma*. For unknown reasons, *toxoplasma*-specific IgM remains detectable until 2 years after the early infection. Similarly, the specific IgA of *Toxoplasma* can last up to 4 years after the primary infection, and then it does not show primary toxoplasmosis. Positive IgM may show an early infection during the period of pregnancy or it may be a reflection of past infections that occurred a few months before the pregnancy. Avidity test is used to differentiate between acute and chronic infection. Avidity is the binding force of antigen to an antibody that its amount in the early stages of antigenicity is low, but in the next months, the amount of avidity is increased by creating B cell antigen (11).

The antigenic contact causes the B cell to mature and more binding between the antigen and antibody, and consequently avidity will occur. High avidity rejects the possibility of acute infection. At now, it seems to combine two methods of IgM ELISA and IgE ELISA are the best and the most reliable results for the diagnosis of acute infection and its differentiation from chronic infection (12). Therefore, this study aimed to evaluate the diagnosis of acute toxoplasmosis by IgG avidity in pregnant women referring to health centers of University in Mashhad.

## Methods

At first, 250 pregnant women who were in the first four months of pregnancy referring to health centers of University in Mashhad (Iranian Academic Center for Education, Culture, and Research {ACECR}, Imam Reza, and Ghaem hospitals) during 2015 were selected after obtaining written consent and completing the checklist of their demographic data.

The study was approved by Ethical Committee of Mashhad University of Medical Sciences with number of IRMUMS.fm.rec.1394.563.

Then, 1 mL of blood sample was collected for detection of toxoplasmosis. After separating the serum from blood samples by Centrifuge at 3000 rpm, sera were stored in –20. Finally, the serum samples were evaluated for the presence of antibodies anti-*Toxoplasma* (IgM and IgG) using direct ELISA (Euroimmun Company) instructions and samples with positive IgM antibody titer were selected and to determine the early and acute infection.

For each IgG and IgM ELISA methods, sera were tested and the amount of cut-off was calculated by this formula: X±2SD (X: the mean of the absorbance, SD: standard deviation of the absorbance for selected samples). The absorbance more and less than the cut off was considered as positive and negative, respectively.

The IgG cut off was as follows:

Negative: proportion <8 IU/mL, borderline: 11 IU/mL > proportion > 8 IU/mL, Positive: proportion> 11 IU/mL. Also, The IgM cut off was as: proportion < 0.8 IU/mL, borderline: 1.1 IU/mL > proportion > 0.8 IU/mL, Positive: proportion> 1.1 IU/mL.

IgG avidity test was used and after adding the avidity buffer and using anti-human IgG and comparing the antibody titer with no adding buffer, Optical Density (OD) was measured at length wave 450 nm. And its index was reported as the percent. Then the results were analyzed using SPSS (ver.16, Chicago, IL, USA) software.

### Inclusion and exclusion criteria

All pregnant women who were in the first four months of pregnancy entered the study. Women with a history of an immunosuppressive disease, *agammaglobulinemia*, kidney transplantation and, malignancy, or people receiving immunosuppressive drugs were excluded.

### Reporting of Relative Avidity Index (RAI)

To calculate the Relative Avidity Index (RAI), the OD Value of the sample washed with urea solution divided by the OD value of the sample washed with PBST solution, multiplied by 100 (according to the kit instructions).

**RAI**< 40%: Antibodies index containing low avidity
**RAI** 40%–60%: Equivocal
**RAI** > 60%: Antibodies index containing high avidity

### Statistical analysis

For data analysis, was used of Frequency, Pearson Chi-Square, Likelihood Ratio, and Fisher Exact Tests. And *P-*value<0.05 was statistically considered as significant.

## Results

The prevalence of anti-toxoplasmosis IgG and IgM in pregnant women referred to the health centers was 23.2% and 7.2%, respectively. Moreover, the highest frequency of IgG and acute toxoplasmosis was observed in the age group of 20–30 yr. accordingly, avidity index of acute toxoplasmosis cases between the pregnant women showed that 62.5%, 6.3% and, 31.3%, respectively had low, intermediate and high avidity. No significant correlation was found between toxoplasmosis IgM and IgG with occupation (*P*=0.83) and (*P*>0.52), respectively. The most cases of acute toxoplasmosis in pregnant women who referred to health centers were in the first month of childbearing and the minimum in the third month. Moreover, no significant relationship was found between IgM and IgG antibodies of *Toxoplasma* and history of contact with the cat (*P*=0.54) and (*P*>0.61), respectively. Table 1 shows the frequency of IgM and IgG antibodies of *Toxoplasma* based on the consumption of uncooked meat in pregnant women who referred to health centers.

**Table 1:** The frequency of IgM and IgG antibodies of *Toxoplasma* based on consumption of uncooked meat in pregnant women

***History of meat uncooked consumption***	***Positive IgG N (%)***	***Positive IgM N (%)***
Positive	2(3.4)	0.0
Negative	56(96.6)	18(100)
Total	58(100)	18(100)
*P*-value	0.39	0.38

Moreover, no significant correlation was found between IgM and IgG antibodies of *Toxoplasma* and consumption of uncooked meat (*P*=0.39) and (*P*>0.38), respectively.

The frequency of IgM and IgG antibodies of *Toxoplasma* based on educational level in pregnant women referred to health centers is presented in Table 2. No significant correlation was found between IgM and IgG antibodies of *Toxoplasma* and educational level (*P*=0.82) and (*P*>0.69), respectively.

**Table 2:** The frequency of IgM and IgG antibodies of *Toxoplasma* based on educational level in pregnant women

***Education***	***Positive IgG N (%)***	***Positive IgM N (%)***
Illiterate	1(5.6)	5(8.6)
Ninth grade	7(38.9)	19(32.8)
Diploma	8(44.4)	25(43.1)
Bachelor and higher	2(11.1)	9(15.5)
*P*-value	0.69	0.82

As shown in Table 3, between the location of residence and IgG antibody of *Toxoplasma* was found a significant correlation (*P*=0.02), but this issue was not observed about IgM (*P*=0.19).

**Table 3:** The frequency of IgM and IgG antibodies of *Toxoplasma* based on place of residence in pregnant women

***Place of residence***	***Positive IgG N (%)***	***Positive IgM N (%)***
Urban life	52(89.7)	18(100)
Rural life	6(10.3)	0.0
Total	58(100)	18(100)
P value	0.02	0.19

## Discussion

*T. gondii* causing toxoplasmosis is an obligate intracellular protozoan parasite that has the potential to infect most warm-blooded vertebrates with the global distribution. Toxoplasmosis in people with a healthy immune system is without clinical symptoms or causes mild clinical symptoms similar to the flu symptoms (13).

Serologic methods in the diagnosis of toxoplasmosis have different sensitivity and specificity and implemented based on the isolation and measurement of antibody using the avidity and affinity. In the present study, there was a significant correlation between age and anti-IgG titer.

In line with our results, in Tehran, Iran (14), and In Ahwaz, southern Iran (15), with increasing age, the seroprevalence of toxoplasmosis increased. In contrast with our findings, in Mashhad (Iran), no significant correlation was observed between age and anti-IgG titer (1).

Based on educational level, the prevalence of anti-*Toxoplasma* IgM and IgG had a significant relationship with education that was in line with the studies conducted in Hamadan, (16) and Khorramabad (17).

In the current study, there was not a significant correlation between the use of raw and semi-cooked meat and IgG and IgM antibodies anti-*Toxoplasma*, which was consistent with other results (17 18), but in contrast with our findings, in Hamadan, a significant correlation was reported between the use of raw and semi-cooked meat and IgG and IgM antibodies anti-*Toxoplasma* (16). In this research, there was no significant correlation between the maintenance of the cat at home and the sero-prevalence of IgG and IgM that was similar to other studies (19–22).

Moreover, there was not a significant relationship between occupation and the prevalence of anti-*toxoplasma* IgG and IgM antibodies, but the prevalence of antibodies in housewives was significantly higher than those employed which was accordant with the findings in France (23).

In the present study, the most prevalence was reported in the city dwellers, but contrary to our study, in England, the highest prevalence was in rural areas (24).

In this study, as for IgG and IgM, the results were similar to another stuy in Tehran (25). In the present study, no significant correlation was found between IgM and IgG antibodies of *Toxoplasma* and consumption of uncooked meat. The undercooked meat is the famous risk factor for toxoplasmosis, although many studies did not report a significant association (26, 27) but in Serbia, this was significant (28). In another retrospective study, about 50% of patients had eaten uncooked meat (29).

Considering the high rate of IgM positive people had a high rate of avidity, we cannot use IgM for the diagnosis of acute toxoplasmosis alone. So, screening for determining the IgM status and also, performing IgG avidity test for the identification of women with acute infection and need for treatment are highly recommended.

## Conclusion

*Toxoplasma* infection should be considered as an important factor that affects the pregnancy and IgG avidity as an important test for screening the women who need the treatment. Moreover, according to a high prevalence of seronegative pregnant women reported in this study and the possibility of acute toxoplasmosis in this group, the preventive measures should be taken.
